# Ptosis Correction: Our Modification and Experience

**DOI:** 10.7759/cureus.26823

**Published:** 2022-07-13

**Authors:** Fahad Hanif, Hasan Tahir, Mirza Shehab A Beg

**Affiliations:** 1 Plastic and Reconstructive Surgery, Liaquat National Hospital and Medical College, Karachi, PAK; 2 Plastic Surgery, Liaquat National Hospital and Medical College, Karachi, PAK

**Keywords:** lagophthalmos, tarsal plate suture, fascia lata, frontalis sling, ptosis

## Abstract

Background

Congenital ptosis not only results in an asymmetric facial appearance but can lead to permanent visual disturbances if not addressed at an appropriate time. Crawford used fascia lata for suspension of the eyelid to frontalis muscle, which remains a standard procedure for congenital ptosis correction to date, with an acceptable recurrence rate due to graft slippage. There are many modifications in this technique to reduce this complication; hence, in this study, we share our experience of a modification to improve the outcomes.

Methodology

This retrospective study was conducted at a private tertiary care hospital in Karachi for 10 years. In total, 26 patients fulfilled our inclusion criteria. All patients underwent a modified Crawford’s procedure under general anesthesia.

Results

In this study, the male-to-female ratio was 1:1.5. In total, 17 (65%) patients had unilateral ptosis. The mean age of presentation was 7 ± 3 years. All of our patients had poor levator function (<5 mm excursion) with a mean of 3 mm and mean grade of ptosis of 4 ± 1.6 mm. The mean preoperative marginal reflex distance (MRD) was +1.8 ± 0.6 mm. In this study, the patients had a mean postoperative MRD of 4.2 ± 0.7 mm at the four-week follow-up.

Conclusions

Although Crawford’s procedure gives promising results for ptosis correction, suturing the fascial sling to the tarsal plate ensures good anchorage and prevents relapse.

## Introduction

Upper eyelid ptosis is both an aesthetic and functional problem that occurs due to weak levator palpebrae superioris activity. Ptosis can be congenital when it is present since birth or can be acquired when it occurs after one year of birth. When the ptosis is congenital the levator muscle has an inherently poor function [1]. Thus, we need to use another motor unit to elevate the upper eyelid. Although there are many procedures to correct ptosis [2-5], the frontalis sling procedure is the gold standard in case of poor levator function [6]. Payr is credited with introducing the use of tensor fascia lata (TFL) as slings for ptosis correction [7]. In 1956, Crawford used fascia lata with frontalis suspension to treat congenital ptosis with poor levator function [8]. Crawford’s technique was a major success as it had good outcomes and reduced the risk of recurrent ptosis. For the past many years, Crawford’s technique has been used with an acceptable rate of sling slippage [9]. However, in 1990, Spoor and Kwitko [10] introduced a technique of direct fixation of the tarsal plate with the frontalis sling. This technique helps to create a definitive lid crease and adjust the lid contour.

In this article, we share our experience of modifying Crawford’s procedure which could prevent postoperative early graft slippage and relapse.

## Materials and methods

This retrospective study was conducted in the Department of Plastic and Reconstructive surgery in a private tertiary care hospital in Karachi, Pakistan. The duration of the study was 10 years (2010-2020). We included patients aged four years and above with poor levator muscle function who were operated on following Crawford’s technique and had intact Bell’s phenomenon. Patients who underwent secondary ptosis correction procedures, had jaw winking phenomenon, or those having preoperative amblyopia or conjunctivitis were excluded from the study.

We retrieved patients’ age, gender, levator function, preoperative and postoperative degree of ptosis using marginal reflex distance (MRD, distance from light reflex to upper eyelid margin), lid crease height, complications in terms of lagophthalmos, recurrence time, signs of exposure keratitis, their satisfaction from the procedure.

Surgical procedure

**Figure 1:**
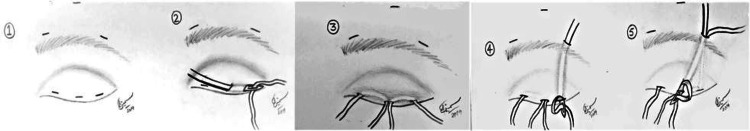
Operative marking (1) and passage of fascial sling through tarsal incisions (2 and 3). Retrieval of the sling from tarsal plate incisions to supra-brow incisions (4 and 5).

**Figure 2:**
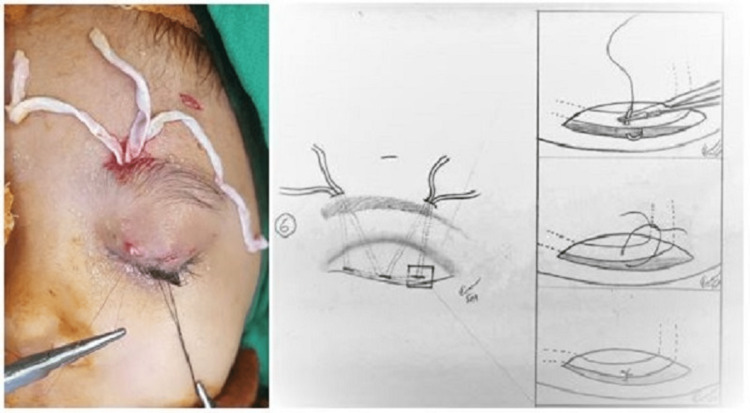
Intraoperative picture (left) and illustration (6) showing suturing of the fascia to the tarsal plate (inset).

**Figure 3:**
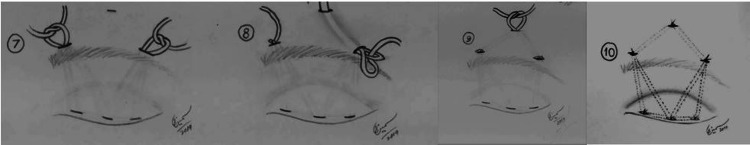
Knot tying after tensioning at the supra-brow (7 and 8) and the mid-forehead (9) incision. Final pentagon format of the procedure (10).

Mark a medial, central, and lateral horizontal skin mark on the eyelid about 2-3 mm from the lash line (Figure 1 - 1 and 2). The skin crease will form here. A higher crease or insertion will reduce the sling’s mechanical advantage in raising the lid. Mark two more incisions vertically just above the eyebrow, one a little lateral to the lateral eyelid mark, and the other a little medial to the medial eye mark. Make a forehead mark above and between these two brow marks to complete an isosceles triangle. We preferably mark and operate on both eyelids at the same time to ensure symmetry. Make stab incisions through all the marks. We use autologous fascia lata to suspend the tarsal plate to the frontalis muscle. Pull one fascial strip from each eyelid incision (Figure 1 - 3) to the eyebrow incision (Figure 1 - 4 and 5) and then through the forehead incision (Figure 2) aiming to make two isosceles triangles.

We stitch fascial slings on tarsal plates with 6-0 absorbable sutures to prevent slippage of fascia lata (Figure 2, inset). A knot is tied using fascial slings which helps to adjust the pull resulting in symmetry of both eyelids, the eyelid reaching the upper limbus, or just leaving the globe (Figure 3). Wounds were closed with 6-0 non-absorbable sutures.

## Results

We operated on a total of 32 patients during the study period; however, 26 patients fulfilled our inclusion criteria. Of these 26 patients, the male-to-female ratio was 1:1.5, with 17 (65%) having unilateral ptosis and nine (35%) having bilateral ptosis. The mean age of presentation was 7 ± 3 years.

All of our patients had poor levator function (<5 mm excursion) with a mean of 3 mm and a mean grade of ptosis of 4 ± 1.6 mm. The mean preoperative MRD was +1.8 ± 0.6 mm, and the mean lid crease distance was 3 mm, with 21 (82%) patients having no lid crease at presentation. Results were comparable in both unilateral and bilateral cases.

Our patients underwent a modified Crawford’s procedure under general anesthesia, as described above. In total, 25 (95%) patients had postoperative lagophthalmos, with no other immediate or late complications (Table 1). One of our patients had focal conjunctivitis on the 10th postoperative day, which was managed with topical medications. The patient had no other symptoms of exposure conjunctivitis. None of our patients had a recurrence of ptosis at a mean follow-up of 20 months; however, two (7.6%) patients required minor readjustment as they were unhappy with the asymmetrical result despite good postoperative MRD. All patients were followed on a weekly basis for a mean follow-up duration of four weeks. Our patients had a mean postoperative MRD of 4.2 ± 0.7 mm at the four-week follow-up.

**Table 1:** Complications among operated patients.

Complications	Number of patients
Lagophthalmos	25
Focal conjunctivitis	1
Recurrence of ptosis	0

All patients were satisfied with the postsurgical outcome, including two patients who needed minor readjustment for slight asymmetry which was addressed accordingly.

## Discussion

Ptosis has several causes, acquired or congenital [11], and when present, it is merely a symptom rather than a diagnosis [6]. Thus, the presence of ptosis requires a thorough clinical evaluation. The amount of levator function generally addresses the choice of surgical approach for correction of ptosis. Based on MRD 1, which is the distance between the upper lid margin and the corneal light reflex (normal MRD 1: 4-5 mm), ptosis is divided into three groups. In unilateral ptosis, the difference in MRD 1 between the two eyes helps classify ptosis as mild ptosis (2 mm), moderate ptosis (3 mm), and severe ptosis (4 mm) [6]. The levator function can be poor (<5 mm), fair (6-9 mm), and good (10-15 mm). Comparable to our results, 68% of the cases were unilateral whereas 32% were bilateral; hence, unilateral cases are more common than bilateral cases. Patients with blepharoptosis undergo surgery at an early age, usually at two to four years of age [12], as it not only leads to an improved cosmesis but it prevents strabismus and amblyopia which can seriously impair visual function [13].

Fascia lata slings and other materials are used for the surgical correction of ptosis. Fascia lata can be harvested from autogenous sources or donor lyophilized or irradiated material can be used. Other materials are also used for frontalis slings which include palmaris tendon graft, deep temporalis fascia graft, marceline mesh [14], and silicone [15]. Fascia lata has excellent biocompatibility when compared to silicone rods which have the benefit of being elastic.

Lid crease incision with tarsal fixation has the benefit of making a secure attachment to the tarsal plate; in addition, it forms a deep lid crease. When present, lash ptosis can be easily corrected with this technique. Utilizing supralash stab incision, there is a minimum disturbance of the lid structures and it does not affect the levator insertion [16]. Crawford’s double triangle method gives a good contour to the lid. The degree of ptosis and the degree of bell’s phenomenon are guides for the adjustment of the height of the operated lid with a frontalis sling. Poor levator and good bell’s phenomenon obviate the need for lids to be raised to a level just below the upper limbus. On the contrary, patients with poor bell’s phenomenon and postoperative lagophthalmos are prone to develop exposure keratopathy; therefore, their lids should be lifted just enough to clear the visual axis. Lagophthalmos is an inherent problem with this procedure, as observed by Young et al. [4]. Moreover, patients with frontalis sling procedure undergo 1.2 ± 1.5 mm lagophthalmos, but it should not produce symptoms of corneal exposure. Generally, younger patients tolerate more lagophthalmos than older patients.

The main limitation of our study is that it is a single-center study. Moreover, a small number of patients were operated on who fit the study criteria. Similar large-scale studies need to be conducted in the future.

## Conclusions

Crawford’s procedure with frontalis sling suspension leads to promising and lasting outcomes. Suturing the fascial sling to the tarsal plate ensures good anchorage and prevents relapse when powerful frontalis works. Therefore, with the results of our study, we conclude that our modification can lead to more promising results with fewer complications.
